# A Novel Algorithm to Quantify Coronary Remodeling Using Inferred Normal Dimensions

**DOI:** 10.5935/abc.20150098

**Published:** 2015-10

**Authors:** Breno A. A. Falcão, João Luiz A. A. Falcão, Gustavo R. Morais, Rafael C. Silva, Augusto C. Lopes, Paulo R. Soares, José Mariani Jr, Roberto Kalil-Filho, Elazer R. Edelman, Pedro A. Lemos

**Affiliations:** 1 Departamento de Cardiologia Intervencionista, Instituto do Coração (InCor), Faculdade de Medicina da Universidade de São Paulo, São Paulo, SP - Brazil; 2 Institute of Medical Engineering and Science, Massachusetts Institute of Technology, Cambridge, MA - USA; 3 Divisão Cardiovascular, Departamento de Medicina, Brigham and Womens Hospital, Harvard Medical School, Boston, MA - USA

**Keywords:** Coronary Artery Diseases, Vascular Remodeling, Atherosclerosis / physiopathology, Neovascularization, Pathologic, Ultrasonography

## Abstract

**Background:**

Vascular remodeling, the dynamic dimensional change in face of stress, can assume different directions as well as magnitudes in atherosclerotic disease. Classical measurements rely on reference to segments at a distance, risking inappropriate comparison between dislike vessel portions.

**Objective:**

to explore a new method for quantifying vessel remodeling, based on the comparison between a given target segment and its inferred normal dimensions.

**Methods:**

Geometric parameters and plaque composition were determined in 67 patients using three-vessel intravascular ultrasound with virtual histology (IVUS-VH). Coronary vessel remodeling at cross-section (n = 27.639) and lesion (n = 618) levels was assessed using classical metrics and a novel analytic algorithm based on the fractional vessel remodeling index (FVRI), which quantifies the total change in arterial wall dimensions related to the estimated normal dimension of the vessel. A prediction model was built to estimate the normal dimension of the vessel for calculation of FVRI.

**Results:**

According to the new algorithm, “Ectatic” remodeling pattern was least common, “Complete compensatory” remodeling was present in approximately half of the instances, and “Negative” and “Incomplete compensatory” remodeling types were detected in the remaining. Compared to a traditional diagnostic scheme, FVRI-based classification seemed to better discriminate plaque composition by IVUS-VH.

**Conclusion:**

Quantitative assessment of coronary remodeling using target segment dimensions offers a promising approach to evaluate the vessel response to plaque growth/regression.

## Introduction

Coronary artery remodeling, the geometric change in artery dimensions, evolves with the ebb and flow of the atherosclerotic process. Arterial remodeling encompasses a wide spectrum of presentations, ranging from expansive to constrictive remodeling^1,2^. In the former, coronary vessel dimensions increase as plaque accumulates, while in the latter there is relative contraction of the vessel wall and impingement on the lumen. There might be a limit to expansive effects, which eventually stabilize or decompensate to luminal encroachment^1^. It is therefore evident that the pattern and extent of arterial remodeling play an important role in ultimately determining the effect of the atherosclerotic disease on luminal dimensions^3-5^.

Several methods have been described to characterize and quantify vessel remodeling in patients with coronary artery disease, mostly using intravascular ultrasound (IVUS) imaging. In cross-sectional studies, the evaluation of coronary remodeling is frequently described as a simple comparison between the most diseased portion and nearby reference segments^6,7^. However, reference vessel segments are not perfect surrogates for normality^8^. In sequential studies a region of interest is examined at baseline and compared with the same matched portion during follow-up^9^. This approach, however, only captures the changes in plaque and vessel dimensions over time, regardless of the degree of atherosclerosis and remodeling at baseline, which may have a marked influence on the outcomes thereafter.

The classification of remodeling varies substantially as a function of definition^10^, and no consensus exists for a universal definition of remodeling^11^. In theory, the ideal method to measure vessel remodeling would evaluate the diseased coronary segment compared to the same region before the existence of the atherosclerotic plaque. Obviously, such a normality comparator cannot be directly assessed in practice. We hypothesized, however, that the native normal vessel size could be inferred for any given coronary segment to create a more appropriate baseline for determination of remodeling. The present study explored a new method to quantify vessel remodeling, based on the comparison between any target segment with its assumed normal dimensions.

## Methods

### Study Design and Patient Population

This prospective, single-arm survey enrolled 67 patients scheduled to undergo coronary angioplasty. During the procedure, before any coronary intervention, all patients were examined with three-vessel coronary IVUS to evaluate coronary geometric parameters. The study was approved by the institutional review board and signed written informed consent was obtained from every patient.

### IVUS Procedure and Image Segmentation

Intracoronary nitroglycerin (100-200 µg) was injected before imaging acquisition. Intravascular ultrasound imaging of the left main trunk and of the proximal portions (40-80 mm) of the three coronary arteries was obtained using a 20 MHz electronic solid-state catheter (Eagle Eye Gold catheter and Vision Gold System console, Volcano Corporation, Rancho Cordova-CA, USA) during automatic pullback at 0.5 mm/s (R100 pullback device, Volcano Corporation, Rancho Cordova-CA, USA).

Two experienced analysts, blinded to clinical data, performed all offline analyses using dedicated software (pcVH 2.2, Volcano Corporation, Rancho Cordova-CA, USA). The external elastic lamina and lumen contours were traced semi-automatically in every acquired IVUS frame to obtain the following grey-scale IVUS parameters: lumen area, elastic external membrane area (EEM area), plaque + media area (EEM area minus lumen area) and plaque burden (plaque + media area divided by the EEM area, multiplied by 100). In addition to the geometric vessel information, radiofrequency analysis of the IVUS signal backscatter, the so-called virtual histology (IVUS-VH), was used to characterize plaque composition into four components: fibrous, fibrolipidic, necrotic core, and dense calcium. The absolute area and percent contribution of each component were computed for all frames.

To verify data accuracy, interobserver reproducibility analyses were performed in 1,000 randomly selected coronary frames of ten patients. The Pearson correlation coefficient for EEM area, lumen area, and plaque + media area were 0.98, 0.95, and 0.93, respectively.

### Calculation of the Novel Fractional Vessel Remodeling Index

The fractional vessel remodeling index (FVRI) was conceived to quantify the total change in arterial wall dimensions related to the atherosclerotic plaque load, and was calculated as:


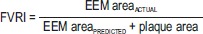


Where,

**EEM area_ACTUAL_** is the real EEM area measured in the cross-section,

**EEM area_PREDICTED_** is the hypothetical dimension of the vessel before the formation of the atherosclerotic plaque (estimated according to the methodology described below), and

**Plaque area** is the current plaque plus media area measured.

For the calculation of the EEM area_**PREDICTED**_, we hypothesized that the original coronary lumen is maintained in the initial phases of the atherosclerotic process. Therefore, all cross-sections with an IVUS plaque burden < 20% were assumed to have normal lumen dimensions. As EEM and lumen areas are coincident on IVUS in the absence of plaque, the estimation of the EEM area_PREDICTED_ was based on the lumen size of cross-sections with absent or trivial plaque (i.e. plaque burden < 20%)^12^. Those cross-sections were analyzed to derive a predictive model for the normal luminal area (i.e. the EEM area_ORIGINAL_) using the following arbitrarily chosen constitutional and anatomical parameters: body surface area, coronary dominance, coronary territory, and the distance in millimeters from the coronary ostium. A final multivariable linear model was built using a bootstrap technique with 5000 replicated samples, with a final prediction equation obtained from the bootstrapped B-coefficients^13^. For the sake of keeping the prediction within the limits of clinically relevant coronary vessels, and because of the sample size, the analysis was restricted to frames with luminal areas between 3.1 mm^2^ and 19.6 mm^2^ (i.e. average vessel diameter between 2.0 mm and 5.0 mm).

### Interpretation of FVRI

**Figure 1:**
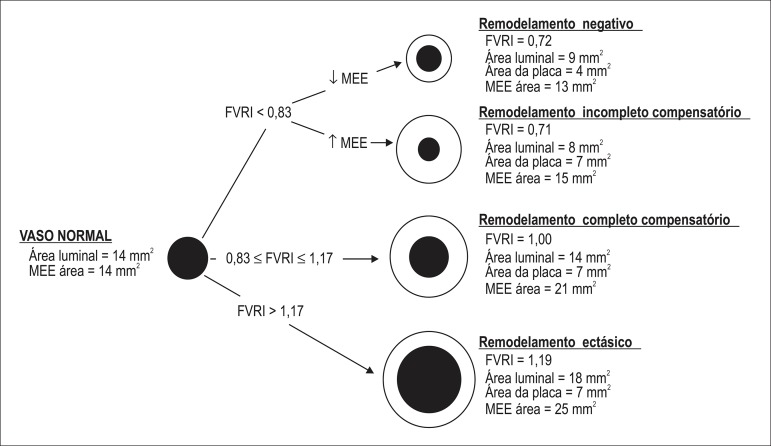
Possible remodeling outcomes of a normal coronary vessel after the occurrence of atherosclerotic plaque. The figure shows the remodeling patterns classified according to the algorithm based on the fractional vessel remodeling index (FVRI). The numeric values are only illustrative. EEM: External elastic membrane.

An FVRI close to a unit, in face of significant plaque, indicates compensatory vessel enlargement resulting in complete accommodation for plaque growth (Figure 1). The cutoff of one standard deviation of FVRI at plaque level was arbitrarily chosen for the FVRI range (between 0.83 and 1.17) to signal “complete compensatory” remodeling. Conversely, an FVRI > 1.17 indicates a disproportionally larger vessel increase compared to the plaque load, denoting "ectatic” remodeling. Finally, an FVRI < 0.83 implies that plaque accumulation was not totally compensated, and there is absolute shrinkage of the vessel (i.e. current EEM is smaller than the hypothetical vessel size) or insufficient vessel enlargement to counterbalance plaque growth.

### Per Cross-Section & Lesion Remodeling Analysis

The FVRI was calculated at the cross-sectional frame level, together with the classification of the remodeling pattern according to the FVRI-based algorithm.

For the lesion level, a coronary lesion was defined as any sequence of three consecutive frames with a plaque burden > 40%^14^. Within each lesion, the frame with the minimal lumen area was chosen as the representative cross-section for the assessment of the lesion remodeling pattern, which was classified according to two methods: the FVRI-based algorithm and the classical remodeling index, calculated as the ratio of EEM area of plaque and reference vessel. In this classic case, EEM plaque area was measured at the in-plaque cross-section with the smallest lumen area, and the reference EEM area was the average EEM area of the proximal and distal references.

The proximal and distal references were specified as the frames with a plaque burden ≤40% adjacent to the respective plaque edges. Only lesions for which both distal and proximal references were available were considered for analysis.

As recently proposed^14^, the plaques were categorized based on the classical remodeling index into "negative remodeling” (classical index < 0.88), "intermediate remodeling" (classical index 0.88 – 1.00) or "positive remodeling" (classical index > 1.00).

### Statistical Considerations

This is an exploratory study for which no formal sample size calculation was performed. A total study population of approximately 65 patients was arbitrarily set to permit, for illustrative purposes, demonstrating a significant linear correlation with an r-coefficient of 0.4 between two continuous variables, considering a two-tailed alpha value of 0.05 and a one-tailed beta value of 0.1^15^. Continuous variables were expressed as mean ± standard deviation and median (interquartile range) and compared by ANOVA one-way testing. Univariable association between continuous variables was assessed by the Pearson correlation method. Categorical variables were expressed by their count and proportions. Statistical significance was set at p < 0.05 and all tests were bicaudal. The regression modelling to estimate the normal vessel size and the calculation of the derived parameters were detailed above. Statistical analyses were performed using SPSS version 21.0 (IBM Corporation).

## Results

Baseline clinical characteristics of the 67 patients (Table 1) display classic demographics of patients presenting for cardiac catheterization and coronary angioplasty. On average, 3.8 ± 1.0 arteries were imaged per patient (total number of coronary arteries = 255): 25% left main, 26% left anterior descending artery, 24% circumflex artery, 22% right coronary artery, 3% others.

**Table 1:** Baseline Characteristics

Age, years	58.9 ± 9.2
Male gender	44 (66%)
Weight, kg	72.0 ± 11.6
Height, cm	161.6 ± 7.9
Body mass index, cm/kg^2^	27.6 ± 4.0
Waist circumference, cm	97.4 + 11.1
Acute coronary syndrome	30 (45%)
Multivessel coronary disease	46 (69%)
Diabetes mellitus	28 (42%)
Hypertension	56 (84%)
Current smoking	14 (21%)
Metabolic syndrome	30 (45%)
Total cholesterol	165.0 ± 39.8
LDL cholesterol	99.9 ± 35.4
HDL cholesterol	36.5 ± 10.3
Triglycerides	143.2 ± 72.1

Numbers are counts (percentages) or mean ± standard deviation.

Overall, 31,159 IVUS cross-sections along a total length of 9,579.8 mm (142.9 ± 22.3 mm per patient) were analyzed. For all frames, lumen area was 8.2 ± 4.0 mm^2^, EEM area was 14.2 ± 5.7 mm^2^, plaque area was 6.0 ± 3.5 mm^2^, and percent plaque burden was 41.6 ± 16.5% of the arterial section. A total of 3,520 cross-sections (11.3%) had no or only mild atherosclerotic plaques (i.e. percent plaque burden < 20%), which were computed for the calculation of the EEM area_PREDICTED_.

**Figure 2:**
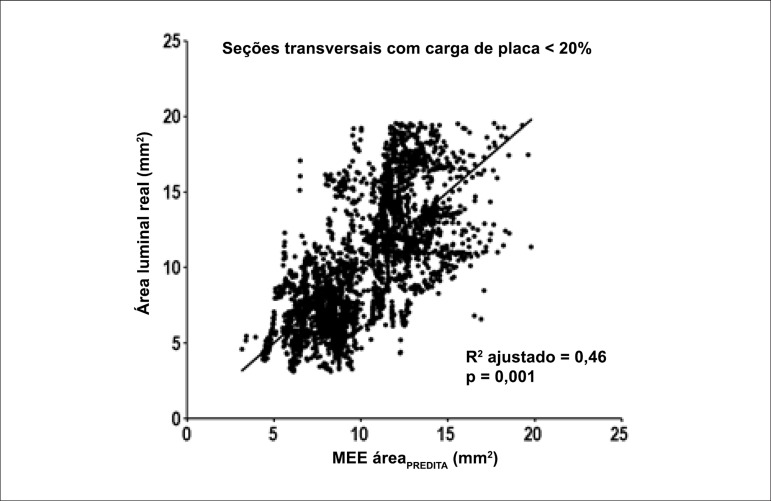
Scatter correlation graph between estimated normal external elastic membrane area (EEM area_PREDICTED_) and the actual lumen area in cross-sections with absent or trivial plaque (plaque burden < 20%).

The overall characteristics of the bootstrapped prediction model to estimate the EEM area_PREDICTED_ (Table 2) demonstrated that all preselected variables remained significant in the final multivariable model. The estimated and the actual vessel areas in cross-sections with absent or trivial plaques (plaque burden < 20%) correlated well (p < 0.001; adjusted R^2^ = 0.46) (Figure 2).

**Table 2:** Final prediction model* to estimate the original external elastic membrane area (EEM area_PREDICTED_)

**Variable**	**Β-coefficient (95% confidence interval)**	**p-value**
Constant	12.20 (11.07 – 13.33)	< 0.001
Dominance pattern	-1.14 (-1.46 – -0.82)	< 0.001
Coronary vessel	-1.73 (-1.80 – -1.66)	< 0.001
Distance from the coronary ostium (in mm) †	-1.28 (-1.39 – -1.18)	< 0.001
Body surface area (in m^2^)	2.60 (1.99 – 3.20)	< 0.001

* Adjusted R^2^ = 0.46;

† Logarithmic transformation.

### Vessel Remodeling at Cross-Section Level

**Figure 3:**
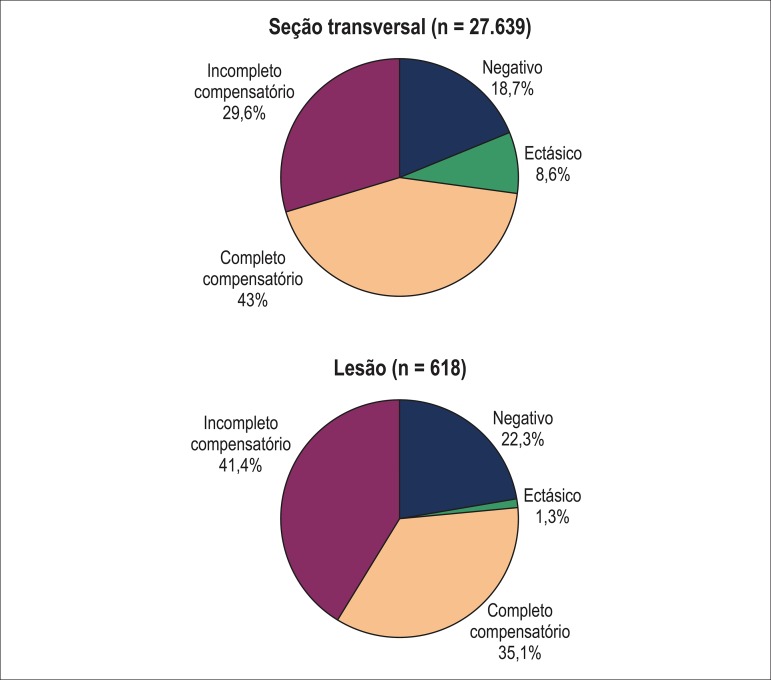
Per cross-section (frames with plaque burden ≥ 20%) and per lesion types of vascular remodeling classified according to the algorithm based on fractional vessel remodeling index (FVRI).

For cross-sections with established plaques (i.e. plaque burden ≥ 20%), the average FVRI was 0.86 ± 0.21 (median 0.84; interquartile range 0.71 – 0.98). Overall, 43% of frames had FVRI between 0.83 and 1.17 ("complete compensatory" remodeling). For the remaining cross-sections, 8.6% had FVRI > 1.17 ("ectatic" remodeling) and, in 48.4%, the FVRI was < 0.83. From these, 38.7% (18.7% of the total) exhibited reduction in EEM area ("negative" remodeling), while 61.3% (29.6% of the total) had insufficient increment in EEM area ("incomplete compensatory" remodeling) (Figure 3).

**Figure 4:**
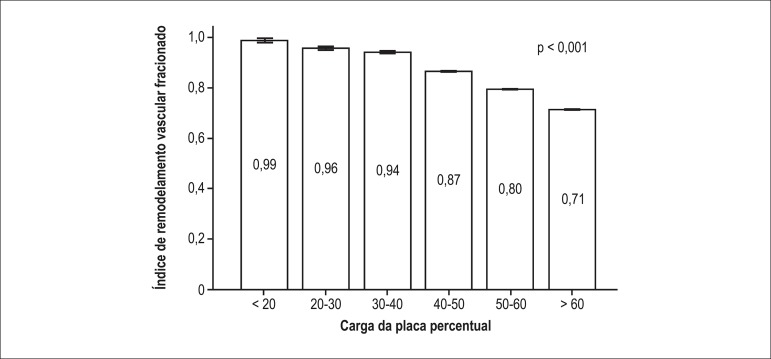
Average fractional vessel remodeling index in relation to percent plaque burden (error bars are one standard error of the mean).

The level of FVRI was influenced by the degree of the atherosclerotic load. FVRI was negatively related to increasing plaque burden (Figure 4); cross-sections with a percent plaque burden < 20% had an average FVRI of 0.99, which progressively decreased to a mean FVRI of 0.71 in frames with plaque burden > 60%.

### Vessel Remodeling at Lesion Level

The analysis included 618 lesions (mean length 7.7 ± 11.2 mm). In-lesion, lumen area was 6.0 ± 3.1 mm^2^, EEM area was 13.4 ± 5.4 mm^2^, and percent plaque burden was 55.0 ± 11.3%. For the mean reference segments, lumen area was 8.9 ± 3.5 mm^2^, EEM area was 14.0 ± 5.4 mm^2^, and percent plaque burden was 36.2 ± 3.2%.

Overall, the in-lesion FVRI was 0.77 ± 0.17 (median 0.77; interquartile range 0.64 – 0.88). When classified according to the FVRI-based algorithm, lesions had complete compensatory remodeling in 35.1%, ectatic remodeling in 1.3%, negative remodeling in 22.3%, and incomplete compensatory remodeling in 41.3% (Figure 3).

The classical remodeling index for the lesions was 0.96 ± 0.16 (median 0.99; interquartile range 0.90 – 1.04). The remodeling categories according to the classical index were: negative remodeling 22%, intermediate remodeling 34.6%, and positive remodeling 43.4%.

The FVRI-based algorithm and the classical remodeling index had a low agreement for the remodeling classification of the lesions, with an overall concordance of only 38.1%: negative/negative in 8.3%, complete compensatory/positive in 17%, and incomplete compensatory/intermediate in 12.8% (Table 3). Nevertheless, there was a significant trend towards increasing FVRI values from negative to positive remodeling categories according to the classical index groups (Table 3).

**Table 3:** Comparative classification of the lesion remodeling patterns according to FVRI-based algorithm or classical remodeling index (n = 618 lesions)

	**Classical remodeling**			Mean classical remodeling index*
	**Negative**	**Intermediate**	**Negative**	
Mean FVRI*	0.70 ± 0.16	0.79 ± 0.18	0.80 ± 0.16	
**FVRI-based remodeling class**				
Negative	51 (8.3)	49 (7.9)	38 (6.1)	0.90 ± 0.16
Incomplete compensatory	57 (9.2)	79 (12.8)	119 (19.3)	0.97 ± 0.18
Complete compensatory	28 (4.5)	84 (13.6)	105 (17.0)	0.99 ± 0.11
Ectatic	0 (0.0)	2 (0.3)	6 (1.0)	1.09 ± 0.15

Numbers are mean ± standard deviation or counts (percentages relative to total number of lesions); FVRI: Fractional vessel remodeling index;

* p< 0.001 for all.

### Impact of Vessel Remodeling on Plaque Composition

**Figure 5:**
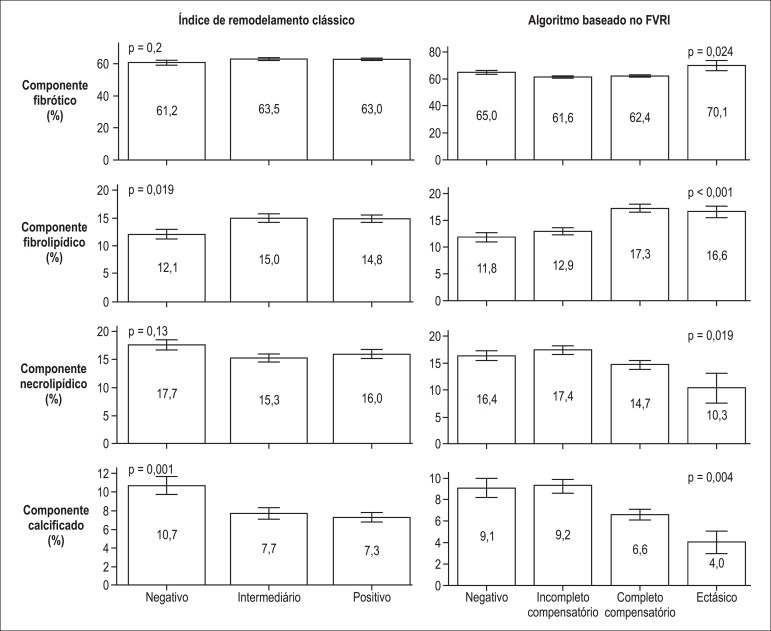
Plaque composition in vascular remodeling types according to fractional vessel remodeling index or classical remodeling index (FVRI) (n = 618 plaques).

The two diagnostic schemes of remodeling classification at plaque level (FVRI or classical remodeling index) were further analyzed for their diagnostic ability in identifying plaque tissue composition. The FVRI-based classification seemed to better discriminate plaque composition: FVRI remodeling classes significantly differed in their plaque composition profile, for all tissue types (fibrous, fibrolipidic, necrolipidic, and calcific) (Figure 5). Conversely, remodeling types by classical remodeling index were not significantly different in relation to their fibrous and necrolipidic components (Figure 5).

## Discussion

Classic quantitative techniques to evaluate coronary remodeling compare vessel size to "normal" adjacent segments, but do so with no standard for distance from the predicate site or precision in "normality". We now describe a new method of assessing coronary vessel remodeling that replaces arbitrary reference vessels with a quantitative approach derived from the estimation of the original normal vessel size. The proposed analytic algorithm, based on the novel FVRI, compares the current vessel to its inferred native state, allowing to measure and classify the remodeling pattern in any point of the coronary tree, providing a numeric assessment of arterial expansion or shrinkage related to coronary atherosclerosis. The proposed method permitted a frame-by-frame, as well as a per-lesion, analysis of the remodeling pattern. To the best of our knowledge, this is the first description of an approach to assess remodeling at individual cross-section level.

The FVRI adds precision and physiologic insight to the remodeling classification, distinguishing vascular responses where plaque is associated with absolute vessel shrinkage from those where plaque growth leads to different degrees of vessel accommodation. In our test population, complete vessel adaptation to plaque accumulation occurred in approximately half of the instances, at both the cross-section and plaque levels. Moreover, partial vessel adaptation to atherosclerosis or negative vessel remodeling (i.e. vessel shrinkage) was often detected, although vessel ectasia was infrequent.

In line with previous studies^12,16^, the present findings indicate that the adaptive vessel enlargement to plaque growth is progressively lost as plaque load increases, beginning as early as plaque burden ~20%, but with a more marked failure in the accommodation in larger plaque burdens. Similar results were seen in a recent cross-sectional substudy from the PROSPECT trial, where compensatory remodeling was also shown to decrease with increasing plaque loads^12^. These results challenge the common concept that lumen dimensions are maintained intact until 40-50% plaque burden occurs^9^.

A number of different approaches have been proposed to measure coronary remodeling in the lesion level^10,14^. Commonly, vessel remodeling is assessed by comparing the vessel size at target segment with the dimensions of adjacent “normal” references^7^. In sequential studies, current guidelines propose the simple change in target vessel size to assess remodeling^17^. Other sequential studies have suggested a classification of remodeling based on the ratio between vessel size and plaque variation^18^. This method, however, is unable to provide quantitative information regarding the magnitude of the remodeling response and is not applicable to segments with minimal or no plaque change (due to division by a null or very low denominator). Use of FVRI reduces some of the caveats of previous methods and may be a viable alternative to quantify remodeling in cross-sectional as well as sequential studies.

A recent study, using alternative cutoff values for the classical remodeling index, showed that "positive" and "negative" remodeling were associated with similar outcomes, and both were worse than "intermediate remodeling"^14^. One could hypothesize that the similarly poorer outcomes for the two opposite types of remodeling, to some extent, might have been related to limitations in measurement and categorization of the remodeling pattern. Indeed, the classical definitions of remodeling as positive, negative, or intermediate are adequate descriptors in only ~40% of cases, as compared to the FVRI-based algorithm. The authors of the previous work reasoned that the impact of remodeling on outcomes could be explained by differences in plaque composition^14^. In line with that, in our series, plaques with classical positive remodeling had more fibrolipidic tissue, while classical negative remodeling was associated with an increase in the calcific component. However, there were no significant differences among the classical remodeling categories in terms of their fibrous and necrotic components. Conversely, the FVRI-based assessment seemed to be more discriminative for the composition of the underlying plaque than the classical approach, with FVRI remodeling types significantly associated with varying profiles for all IVUS-VH plaque components. Altogether, FRVI appears to stratify coronary remodeling into four, instead of three, physiologically meaningful patterns with markedly different plaque composition. Whether these findings will be translated to the addition of clinical value by FVRI assessment remains open for future investigations.

Our analyses suggest that the estimation of the original normal lumen and vessel size in any point of the coronary tree - a crucial step to calculate FVRI - is feasible and easily obtained. Nevertheless, due to the relatively small sample size of the present study and the intrinsic statistical limitations of any prediction modeling of multiple interdependent parameters, future studies are warranted to further refine and validate the estimation of normal vessel dimensions. It must be highlighted, however, that our study does not aim at validating the proposed method, but has the main objective of describing the theoretical concept of the FVRI-based algorithm for remodeling assessment and providing initial exploratory results of the new score.

## Conclusion

The FVRI provides a quantitative assessment of coronary vessel remodeling, independent of nearby references, and offers a promising approach to evaluate the vessel response to plaque growth/regression.
